# The frequency of radial artery occlusion following cardiac catheterization with the use of transradial pneumatic compression band

**DOI:** 10.1186/s13104-020-05323-8

**Published:** 2020-10-20

**Authors:** Kashif Ali Hashmi, Zahid Iqbal, Atif Ali Hashmi, Maira Shoaib, Muhammad Irfan, Rabeet Khan, Nauman Ali, Amir khan, Muhammad M. Edhi

**Affiliations:** 1 Chaudhry Pervaiz Elahi Institute of Cardiology, Multan, Pakistan; 2 Liaquat National Hospital and Medical College, Karachi, Pakistan; 3 Oxford University Hospitals NHS Trust, Oxford, UK; 4 Kandahar University, Kandahar, Afghanistan; 5 Brown University, Providence, RI USA

**Keywords:** Cardiac catheterization, Radial artery occlusion, Thrombosis, Transradial pneumatic pressure band

## Abstract

**Objectives:**

Radial artery occlusion is a silent complication of a transradial approach to cardiac catheterization that may complicate subsequent transradial procedures in patients undergoing cardiac catheterization. A transradial band reduces vascular complications and provides brisk, powerful and effective haemostasis. The purpose of this study was to assess the frequency of radial artery occlusion in 180 patients undergoing transradial coronary catheterization.

**Results:**

The median age of the study cohort was 58 years. Radial artery occlusion was found in 14 (7.8%) patients. When stratifying by age group and sex, there was no significant difference in radial artery occlusion between age groups and sex. It was likewise found that comorbidities such as diabetes mellitus, hypertension and smoking, increased the risk of radial artery occlusion however this was observed to be significant only for diabetes mellitus. We therefore conclude that a transradial pneumatic pressure band is an extremely helpful and safe strategy to prevent radial artery occlusion.

## Introduction

Coronary angiography is viewed as the most effective technique for the diagnosis and management of ischemic heart disease (IHD) [1]. The two most common routes for cardiac catheterization are femoral and radial. Current literature demonstrates that radial access is more secure than femoral access. In any case, the most widely recognized benefit of utilizing a transradial approach is the lower rate of vascular complications [2]. Complications of a radial approach include radial artery spasm, radial artery occlusion, and rarely hematoma or perforation [3].

Radial artery occlusion (RAO) is typically a silent complication of the transradial approach to cardiac catheterization and it may increase complication rates in future transradialcatheterizations. Plethysmography and duplex ultrasound are required for the diagnosis of RAO as palpation of the pulse at the site of cannulation is not always dependable [4]. An investigation that compared the reliability and viability of a transradial band versus radistop hemostatic pressure gadgets after transradial coronary mediation, demonstrated that RAO was present in 9.2% of patients at the time of discharge and 6.8% of patients at the time of follow-up [5]. Radical access is valuable to patients as it permits early ambulation. Although it is more practical than the femoral approach; it is associated with a higher rate (5–10%) of asymptomatic radial artery occlusion [6]. In third world countries like Pakistan, complication rates of cardiovascular diseases are quite high [7–10] and there is need to utilize effective techniques that can reduce complication rates.

Transradial (TR) band utilization is a reliable technique that produces quick hemostasis. A TR band is composed of a plate alongside two inflatable balloons. It reduces the risk of RAO. Maintaining radial artery patency through pressure application also prevents future RAOs [11].

The objective of this study was to assess the effectiveness of using a TR band in reducing the risk of RAO when used after a coronary angiogram. The effectiveness of TR band utilization has been recognized internationally [11–13] but data is still lacking in our region.

## Main text

### Methods

This study included a total of 180 male and female patients undergoing a coronary angiogram via transradial access at the Ch. Pervaiz Elahi Institute of Cardiology Multan between January 2016 and December 2017. The age range of the study group was 18–70 years. Patients who had undergone previous coronary angiograms through transradial access were excluded from the study. After approval from the hospital ethics committee, all patients satisfying the inclusion criteria were incorporated into the study. Informed consent was taken from patients for utilizing their information in research. Clinically relevant details including age and sex were incorporated into the study and the impact of hypertension (HTN), diabetes (DM) and smoking on the results of the investigation were analyzed. HTN was assessed based on a past medical history (PMH) of HTN or the patient having a blood pressure of > 140/90 mmHg on examination. DM was assessed either through PMH, a fasting glucose > 126 mg/dl or a random blood glucose > 200 mg/dl. Smoking history was determined from the patient's social history. The radial artery occlusion (RAO) was evaluated by Barbeau test 24 h post coronary angiography. Barbeau test was performed by a consultant cardiologist with 5 years of experience.

### Data analysis

Data was evaluated using a statistical package for social sciences (SPSS) version 21. Mean and standard deviation was recorded for quantitative variables while frequency and percentage were determined for qualitative factors. Stratification was conducted to assess the impact of modifiers on study groups by utilizing the chi square test. Fisher exact test was applied where expected numbers were small (i.e. < 5 cells). A P value ≤ 0.05 was considered significant.

### Results

**Fig. 1:**
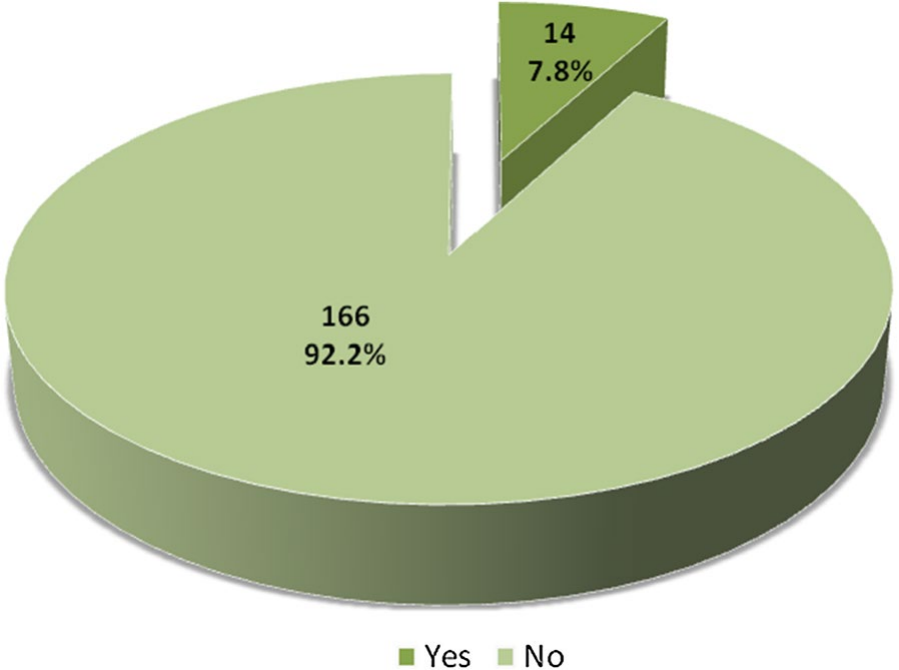
Radial artery occlusion among study population

Out of 180 patients, 123(68.3%) were male and 57(31.6%) were female. Median age of the patients was 58 years, ranging from 18 to 70 years. Among 180 patients, 82(46.1%) patients were found to have diabetes mellitus and 91(50.5%) had hypertension. 102 (56.7%) patients were smokers (as presented in Table 1). In our study, 14 (7.8%) patients were found to have radial artery occlusion as presented in Fig. 1.

**Table 1:** Descriptive statistics of study population

	n (%)
Age, median (range)	58 (18–70)
Gender	
Male	123 (68.3)
Female	57 (31.6)
Diabetes mellitus	
Present	83 (46.1)
Absent	97 (53.9)
Hypertension	
Present	91 (50.5)
Absent	89 (49.5)
Smoking	
Yes	102 (56.7)
No	78 (43.3)

Stratification was conducted and the chi square test was applied. The results show insignificant association of radial artery occlusion with diabetes mellitus (p = 0.048) and an insignificant association with gender (p = 0.768), age group (p = 0.526), hypertension (p = 0.608) and smoking (p = 0.085). The results are presented in Table 2.

**Table 2:** Association of Radial artery occlusion with clinical features and co-morbids

	Radial artery occlusion n (%)		P-value
	Present (n = 14)	Absent (n = 166)	
Gender			
Male	9 (64.3)	114 (68.7)	0.768↨
Female	5 (35.7)	52 (31.3)	
Age group			
≤ 40 years	1 (7.1)	29 (17.5)	0.526↨
41–60 years	6 (42.9)	78 (47)	
> 60 years	7 (50)	59 (35.5)	
Diabetes mellitus			
Present	10 (71.4)	73 (44)	0.048
Absent	4 (28.6)	93 (56)	
Hypertension			
Present	8 (57.1)	83 (50)	0.608
Absent	6 (42.9)	83 (50)	
Smoking			
Yes	11 (78.6)	91 (54.8)	0.085
No	3 (21.4)	75 (45.2)	

Chi-square test was applied

↨Fisher exact test was applied

P-value ≤ 0.05, considered as significant

### Discussion

In this analysis, we found that only 7.8% of patients developed radial artery occlusion with utilization of a transradial band. Rathore et al. [5] conducted a randomized correlation of TR band and radistop hemostatic pressure gadgets after transradial coronary mediation. He showed that radial artery occlusion at the time of discharge was seen in 9.2% of patients and 6.8% of patients demonstrated occlusion even at the time of follow-up. In another investigation, Pancholy et al. [13] assessed the impact of two distinctive hemostatic gadgets (HemoBand and Inflatable TR Band) on radial artery occlusion after transradial catheterization. In the Hemoband group, 11.2% of patients developed occlusion within 24 h, compared with 4.4% in the Inflatable TR band group (p < 0.005). In the Hemoband group, 7.2% patients developed occlusion (at 30 days), whereas 3.2% patients developed occlusion with the Inflatable TR band. (p < 0.05). A considerable decrease in radial artery occlusion was noted with hemostasis when utilizing the TR Band in contrast with the Hemo Band.

The incidence of RAO has been reduced in recent years because of the more utilization of radial approach. In a study by catheter laboratory, the incidence of RAO was 15% in a randomly selected group of 352 patients [14].

Zankl et al. [15] detected RAO by Doppler in 10.5% patients undergoing coronary angioplasty. The number of symptomatic patients in this cohort presenting with lower arm pain was 58.5% however none of these patients had symptomatic hand ischemia. Treatment with low molecular weight heparin (LMWH) for duration of 1 month caused arterial recanalization in 86.7% of these patients and also alleviated side effects. In another examination, 42.5% of patients with RAO reported pain in the forearm within 24 h following the transradial coronary procedure, with another 7% of patients presenting with similar symptoms a few days later. There was no indication of acute limb ischemia in any patient. Fifty-nine percent of patients with RAO were treated with LMWH. Arterial recanalization, evaluated following 14 days, was significantly higher in the LMWH treated group compared to the group without anticoagulative treatment (55.6% versus 13.5%, p < 0.001) [11].

The Prevention of Radial Artery Occlusion-Patent Hemostasis Evaluation Trial (PROPHET) investigated the effectiveness of patent hemostasis using the Hemoband (HemoBand Corporation, Portland, OR) [11]. Patients were randomly allocated to either a conventional pressure application for haemostasis group (occulusive haemostasis technique) or a pressure application guided by heart beat oximetry to confirm patent haemostasis group (the ulnar artery was blocked and the HemoBand was released until a pulsatile plethysmography sign was observed). The patent haemostasis group had altogether less RAO than the control group, both at 24 h (5% versus 12%, p < 0.05) and at 1 month (1.8% versus 7.0%, p < 0.05). Consequently, it was suggested that the TR pneumatic pressure band is an extremely helpful and safe technique in diminishing the risk of radial artery occlusion after transradial cardiovascular catheterization.

## Limitations

The key limitation of this investigation was that data collection was only conducted at a single institution. A multicenter study would be useful in determining whether the radial artery occlusion rate of 7.8% is consistent with other medical centers in the region. Secondly, the sample size was small; therefore the study was under-powered to draw any meaningful conclusions regarding association with other clinical parameters and co-morbids. Based on our results, we therefore conclude that a transradial pneumatic pressure band is an extremely helpful and safe strategy to prevent radial artery occlusion after cardiac catheterization.

## Data Availability

The datasets used during this study are available from the corresponding author on request.

## Abbreviations

RAO: Radial artery occlusion
IHD: Ischemic heart disease

## References

[1] Grossman W, Baim DS (2000). Grossman's cardiac catheterization, angiography, and intervention.

[2] Jolly SS, Yusuf S, Cairns J, Niemelä K, Xavier D, Widimsky P (2011). Radial versus femoral access for coronary angiography and intervention in patients with acute coronary syndromes (RIVAL): a randomised, parallel group, multicentre trial. Lancet.

[3] Dandekar VK, Vidovich MI, Shroff AR (2012). Complications of transradial catheterization. Cardiovasc Revasc Med.

[4] Brancati MF, Burzotta F, Coluccia V, Trani C (2012). The occurrence of radial artery occlusion following catheterization. Expert Rev Cardiovasc Ther.

[5] Rathore S, Stables RH, Pauriah M, Hakeem A, Mills JD, Palmer ND (2010). A randomized comparison of TR band and radistop hemostatic compression devices after transradial coronary intervention. Catheter Cardiovasc Interv.

[6] Cubero JM, Lombardo J, Pedrosa C, Diaz-Bejarano D, Sanchez B, Fernandez V (2009). Radial compression guided by mean artery pressure versus standard compression with a pneumatic device (RACOMAP). Catheter Cardiovasc Interv.

[7] Hashmi KA, Abbas K, Hashmi AA (2018). In-hospital mortality of patients with cardiogenic shock after acute myocardial infarction; impact of early revascularization. BMC Res Notes.

[8] Hashmi KA, Shehzad A, Hashmi AA, Khan A (2018). Atrioventricular block after acute myocardial infarction and its association with other clinical parameters in Pakistani patients: an institutional perspective. BMC Res Notes.

[9] Hashmi KA, Saeed HY, Ahmed J, Najam J, Irfan M, Hashmi AA (2020). Left ventricular thrombus formation in acute anterior wall myocardial infarction: a comparison between thrombolyzed and non-thrombolyzed patients. Cureus.

[10] Hashmi KA, Saeed HY, Farid M (2020). Frequency of multivessel severe coronary artery disease in patients with non-ST segment elevation myocardial infarction having markedly raised cardiac troponin T. Cureus.

[11] Pancholy S, Coppola J, Patel T, Roke-Thomas M (2008). Prevention of radial artery occlusion-patent hemostasis evaluation trial (PROPHET study): a randomized comparison of traditional versus patency documented hemostasis after transradial catheterization. Catheter Cardiovasc Interv.

[12] Lombardo-Martínez J, Díaz-Bejarano D, Pedrosa-Carrera C, Sánchez-Baños B, Gómez-Santana C, Fernández Alvarez V (2009). Clinical trial of radial artery compression guided by mean arterial pressure. Enferm Clin..

[13] Pancholy SB (2009). Impact of two different hemostatic devices on radial artery outcomes after transradial catheterization. J Invasive Cardiol.

[14] Sławin J, Kubler P, Szczepański A, Piątek J, Stępkowski M, Reczuch K (2013). Radial artery occlusion after percutaneous coronary interventions—an underestimated issue. PostepyKardiolInterwencyjnej.

[15] Zankl AR, Andrassy M, Volz C, Ivandic B, Krumsdorf U, Katus HA, Blessing E (2010). Radial artery thrombosis following transradial coronary angiography: incidence and rationale for treatment of symptomatic patients with low-molecular-weight heparins. Clin Res Cardiol..

